# Optimization of culture conditions for the expression of three different insoluble proteins in *Escherichia coli*

**DOI:** 10.1038/s41598-019-53200-7

**Published:** 2019-11-14

**Authors:** Matías Gutiérrez-González, Camila Farías, Samantha Tello, Diana Pérez-Etcheverry, Alfonso Romero, Roberto Zúñiga, Carolina H. Ribeiro, Carmen Lorenzo-Ferreiro, María Carmen Molina

**Affiliations:** 1 Centro de Inmunobiotecnología, Programa Disciplinario de Inmunología, Instituto de Ciencias Biomédicas, Facultad de Medicina, Universidad de Chile, Santiago, Chile; 2 Programa de Doctorado en Farmacología, Facultad de Ciencias Químicas y Farmacéuticas, Universidad de Chile, Santiago, Chile; 3 Área de Biotecnología, Instituto Polo Tecnológico de Pando, Facultad de Química, Universidad de la República Oriental del Uruguay, Montevideo, Uruguay

**Keywords:** Microbiology techniques, Expression systems

## Abstract

Recombinant protein expression for structural and therapeutic applications requires the use of systems with high expression yields. *Escherichia coli* is considered the workhorse for this purpose, given its fast growth rate and feasible manipulation. However, bacterial inclusion body formation remains a challenge for further protein purification. We analyzed and optimized the expression conditions for three different proteins: an anti-MICA scFv, MICA, and p19 subunit of IL-23. We used a response surface methodology based on a three-level Box-Behnken design, which included three factors: post-induction temperature, post-induction time and IPTG concentration. Comparing this information with soluble protein data in a principal component analysis revealed that insoluble and soluble proteins have different optimal conditions for post-induction temperature, post-induction time, IPTG concentration and in amino acid sequence features. Finally, we optimized the refolding conditions of the least expressed protein, anti-MICA scFv, using a fast dilution protocol with different additives, obtaining soluble and active scFv for binding assays. These results allowed us to obtain higher yields of proteins expressed in inclusion bodies. Further studies using the system proposed in this study may lead to the identification of optimal environmental factors for a given protein sequence, favoring the acceleration of bioprocess development and structural studies.

## Introduction

It is well established that systems of high recombinant protein expression levels are required for structural studies and therapeutic uses. Biological expression systems that are currently used include: prokaryotic, plant-based and eukaryotic expression systems, each with well-known advantages and disadvantages^1–3^. Among prokaryotic expression systems, *Escherichia coli* remains the workhorse for several applications, given its fast growth, high densities achieved and feasible manipulation^1^. However, not all proteins are efficiently produced in this system, as low solubility of the target protein and subsequent inclusion bodies (IB) formation may restrict its successful application^3^. Several strategies have been developed to overcome this undesirable limitation, which target environmental parameters, such as culture temperature or inducer concentration, as well as intrinsic protein variables, such as relative codon abundance or fusion to more soluble proteins^4^. However, there is no “one size fits all” strategy *a priori* to obtain an active, soluble protein and, as a consequence, empirical observations for each protein is needed, which can be both costly and time consuming.

In some situations, the accumulation of recombinant protein in IBs is unavoidable, and it represents a challenging condition when recombinant proteins are needed in a fast and reliable fashion. The technical procedures to obtain soluble and active proteins from IBs are labor intensive and require a combination of rational and empirical knowledge. In this sense, a valuable approach would be to increase the yield of recombinant protein in this state, as IBs can, in fact, protect the recombinant protein from proteolytic degradation and prevent the bacteria from recombinant protein toxicity. With several batches of correctly stored IBs, a researcher can explore some alternatives to obtain a final, soluble protein preparation^5^.

Bioprocess improvement can be achieved by changing one factor at a time (OFAT). However, although attractively simple, this is a limited methodology, given the complex nature of the determinants of protein expression, solubility and folding. In this scenario, OFAT is not the most efficient approach to obtain information on the operation space, as changing one input can have unexpected effects on the outcomes of other, unrelated, variables^6^. The Design of Experiments (DoE) methodology is a more appropriate approach, as it requires less resources and systematizes interaction discovery. Importantly, there are several DoE settings, each with its own advantages and disadvantages. Three-level Box-Behnken methods are a type of incomplete factorial designs, with slightly more efficiency than Central Composite Designs and much more effective than full factorial designs^7^. The application of this methodology results in less experiments aiming to obtain the coefficients for the estimated model.

MHC class I chain–related protein A (MICA) is a transmembrane protein expressed as a result of cellular stress. NKG2D receptor, present on the surface of natural killer and cytotoxic cells, can recognize MICA and trigger target cell lysis. However, tumor cells can escape this immunosurveillance mechanism by expressing a soluble form of MICA, which downregulates NKG2D expression on effector cells. Moreover, it has been observed that high serum levels of MICA are correlated with disease progression in a variety of human cancers^8^. This led us to develop a single chain variable antibody (scFv), isolated from a phage display library, directed against the recognition interface between MICA and NKG2D; by preventing MICA-mediated NKG2D downregulation, this scFv could potentially serve as therapy in MICA expressing cancers^9^. scFvs are composed of variable regions from heavy and light chains from immunoglobulins, and fused with a flexible linker. This protein format can be expressed in *E. coli*, and direct modification of its amino acidic sequence can be carried out for affinity maturation^10^.

IL-23 is a heterodimeric protein member of the IL-12 cytokine family, sharing with this last cytokine the p40 subunit^11,12^. The p19 subunit, on the other hand, is unique to IL-23, and it is an interesting therapeutic target, as IL-23 has been linked to immune-related diseases, such as Crohn’s disease and psoriasis^13–18^. Thus, expression of this protein at large scales is attractive for the development of new and effective treatments for these diseases.

Here, we present the analysis and optimization of the expression conditions for three different proteins, a anti-MICA scFv, MICA, and p19 subunit of IL-23, which are expressed as insoluble recombinant proteins in *E. coli*. We favored speed of analysis using a three-level Box-Behnken design, with post-induction temperature, post-induction time and IPTG (Isopropyl β-D-1-thiogalactopyranoside) concentration as factors, generating 15 experimental runs. The resulting models allowed us to obtain the optimum environmental variables for each protein, and to compare the behavior of these insoluble proteins with data from soluble proteins available in the literature. We further optimized protein refolding conditions, which resulted in the generation of soluble and active scFv for binding assays. We also performed a multivariate analysis of the sequence-derived features and optimal environmental variables for protein expression and compared soluble and insoluble proteins, which revealed important differences in terms of favorable environmental variables and amino acid sequence features.

## Results

### anti-MICA scFv, MICA and IL-23p19 are expressed as inclusion bodies in *E. coli*

**Figure 1:**
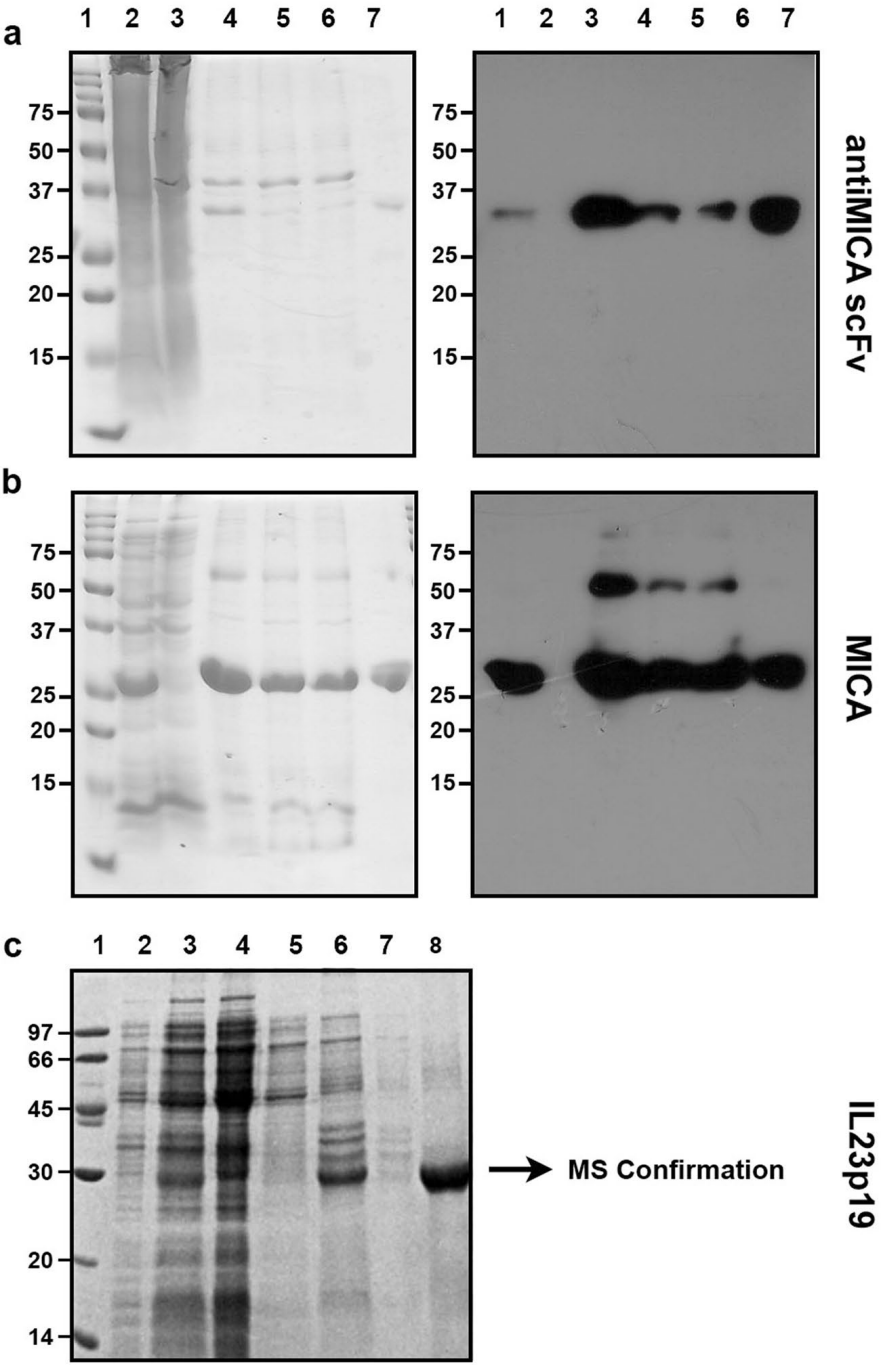
anti-MICA scFv, MICA , and IL-23-p19 are expressed in inclusion bodies in *E. coli* BL21. Samples from different culture steps were separated by SDS-PAGE (left), and analyzed by western blot (right) using an anti-His antibody. (**a**) anti-MICA scFv. (**b**) MICA. (**c**) IL-23p19. Molecular weights are indicated in kDa. 1: Protein ladder. 2: Total bacterial lysate after induction. 3: Soluble fraction of lysate. 4: Insoluble fraction of lysate. 5: Flow through of chromatographic purifications. 6: Elution. For C: 1: Protein ladder. 2: Total bacterial lysate before induction. 3: Total bacterial lysate after induction. 4: Soluble fraction of lysate. 5: Soluble fraction after incubation with Triton X-100. 6: Soluble fraction after treatement with N-Lauroylsarcosine. 7: Flow through of chromatographic purifications. 8: Elution. The bands of expected size were analyzed by mass spectrometry (MALDI-TOF/TOF 4800 Analyzer, Applied Biosystems, Framingham, USA) confirming the identity of interleukin-23 subunit alpha precursor *Homo sapiens* (data not shown).

A low yield of soluble proteins was obtained when MICA, anti-MICA scFv and IL-23p19 were expressed in *E. coli*, whereas proteins in inclusion bodies represented more than 90% of recombinant proteins (Fig. 1). Attempts in protocol optimization, including changes in culture temperature, IPTG concentration or induction time were unsuccessful to obtain soluble proteins. However, we could effectively purify these proteins from inclusion bodies (data not shown), and decided to optimize protein expression from this compartment. Optimization was first carried out by selecting the most appropriate operating space for each protein. For MICA and anti-MICA scFv, previous experiments showed that the operating space lies between 3–6 h (post-induction time) and 0.1–1 mM IPTG, which were used in the present designs. In the case of IL-23p19, a range of 3–5 h (post-induction time) and 0.2–1 mM IPTG was applied for protein expression.

### Optimization of anti-MICA scFv, MICA and IL-23p19 production in inclusion bodies in *E. coli* using response surface methodology

We developed a model to determine optimal bacterial culture temperature, IPTG concentration and post-induction time using response surface methodology (RSM). This technique is useful when a response of interest, in this case protein concentration, is dependent on several independent factors^19^. The design matrix was generated using RcmdrPlugin.DoE in a R environment. Table 1 shows detailed information on the design matrix, including coded and actual variables for each run. For each protein, the expression values obtained were analyzed by RSM, which retrieved the following equations:

1 $$\begin{array}{ccc}Y[scFv(\mu g/mL)] & = & 0.179-0.022A+0.014B+0.012C-0.049{A}^{2}\\  &  & -0.018{B}^{2}-0.039{C}^{2}\end{array}$$

2 $$Y[MICA(\mu g/mL)]=0.315+0.145A+0.087B-0.068C$$

3 $$\begin{array}{ccc}Y[IL23-p19(\mu g/mL)] & = & 2.5+1.125A-0.750B+0.025C-0.375AB\\  &  & -0.25AC-0.175BC\,+\,0.762{A}^{2}-0.588{B}^{2}-0.188{C}^{2}\end{array}$$

where Y is the response variable (protein in µg/mL, in eluate), A refers to post-induction temperature, B refers to post-induction time, and C represents IPTG concentration.

**Table 1:** Box-Behnken design.

Variable	Level		
Coded values	−1	0	+1
A: Temperature (°C)	25	31	37
B: Time (h)	3	4,5	6
C: IPTG (mM)	0.1 (MICA, scFv), 0.2 (IL-23p19)	0.55 (MICA, scFv), 0.6 (IL-23p19)	1

Coded and actual variables are shown. For time and IPTG, information shown is for MICA, anti-MICA scFv (left) and IL-23p19 (right).

**Figure 2:**
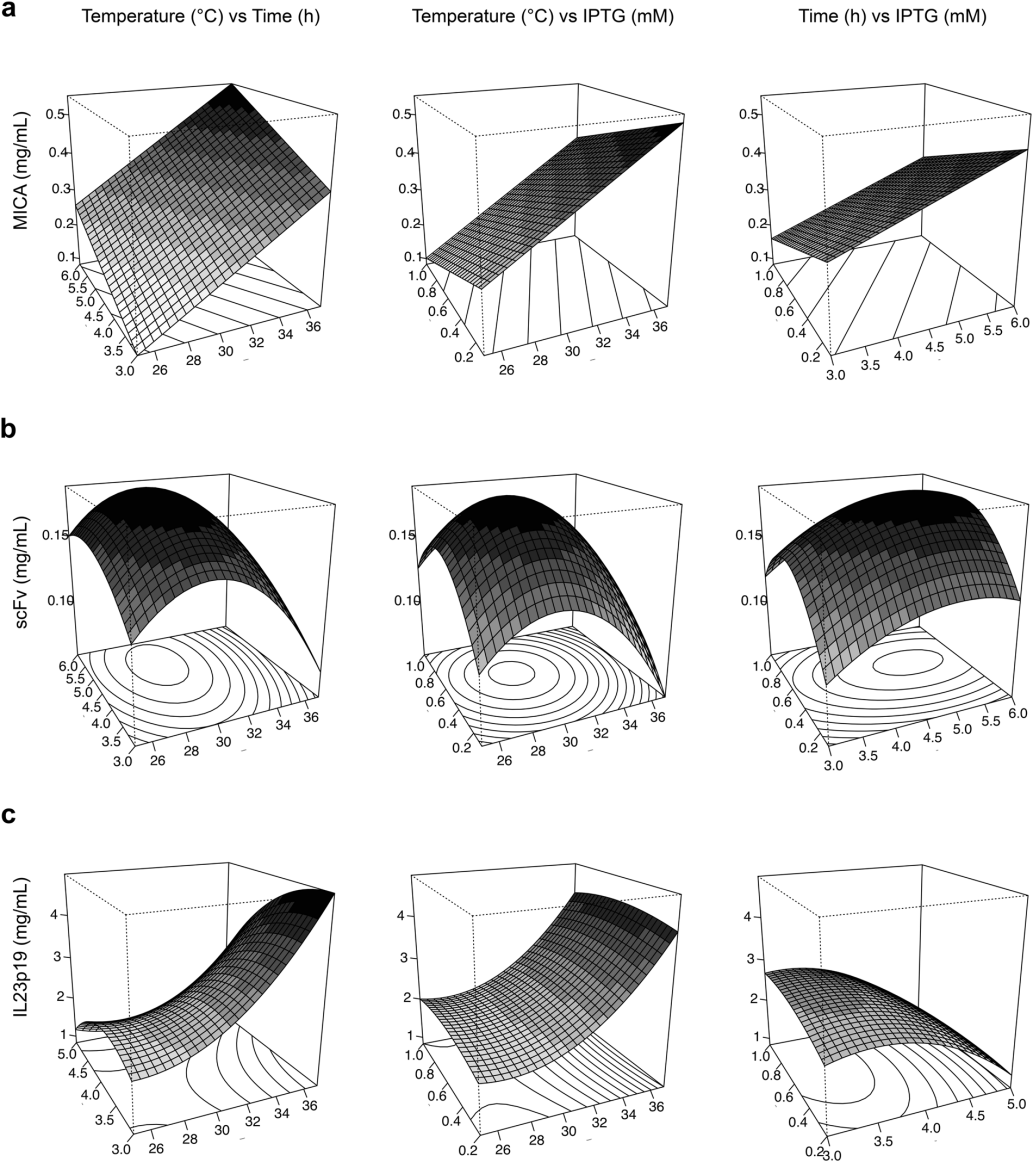
Surface response plots of expressed proteins. The effect of post-induction temperature, post-induction time and IPTG concentration on the expression of MICA (**a**), anti-MICA scFv (**b**) and IL-23p19 (**c**) is shown.

The yield for each protein varied significantly, as displayed in Table 2, with IL-23p19 showing the greatest protein concentration. More importantly, every protein data point was fitted with different models and particular optimal conditions could be determined. The model obtained for scFv expression (r^2^ = 0.7524, p = 0.036) shows that the most significant variables were post-induction temperature and IPTG concentration, both in their quadratic forms. In the case of MICA, a first order model (r^2^ = 0.8262, p = 1.72 × 10^−4^) was selected, in which the three variables showed a high impact in the observed yields. Finally, in the case of IL-23p19, a complete second order model (r^2^ = 0.9773, p = 1.36 × 10^−3^) indicated that the most significant variables were temperature and time, both in first order and quadratic forms. All fitted models are shown in Fig. 2. Based on the normal Q-Q plot shown in Fig. S1, we concluded that the residuals are normally distributed in the three models generated.

**Table 2:** Experimental runs for Box-Behnken design.

Run	Coded			Actual			Yield (mg/mL)		
	A	B	C	Temperature (°C)	Time (h)	IPTG (mM)	MICA	anti-MICA scFv	IL-23p19
1	−1	−1	0	25	3	0.55/0.6	0.068	0.11	1.8
2	1	−1	0	37	3	0.55/0.6	0.285	0.094	5.1
3	−1	1	0	25	6/5	0.55/0.6	0.249	0.177	1
4	1	1	0	37	6/4	0.55/0.6	0.633	0.067	2.8
5	−1	0	−1	25	4.5/4	0.1/0.2	0.311	0.077	2.2
6	1	0	−1	37	4.5/4	0.1/0.2	0.563	0.085	4.2
7	−1	0	1	25	4.5/4	1	0.103	0.133	2
8	1	0	1	37	4.5/4	1	0.415	0.068	3.9
9	0	−1	−1	31	3	0.1/0.2	0.249	0.083	2.1
10	0	1	−1	31	6/5	0.1/0.2	0.426	0.133	1
11	0	−1	1	31	3	1	0.248	0.123	2.8
12	0	1	1	31	6/5	1	0.237	0.147	1
13	0	0	0	31	4.5/4	0.55/0.6	0.221	0.154	2.5
14	0	0	0	31	4.5/4	0.55/0.6	0.339	0.164	2.7
15	0	0	0	31	4.5/4	0.55/0.6	0.317	0.220	2.3

Coded and actual variables are shown. For time and IPTG, information shown is for MICA, anti-MICA scFv (left) and IL-23p19 (right).

### Multivariate analysis of soluble and insoluble proteins

**Figure 3:**
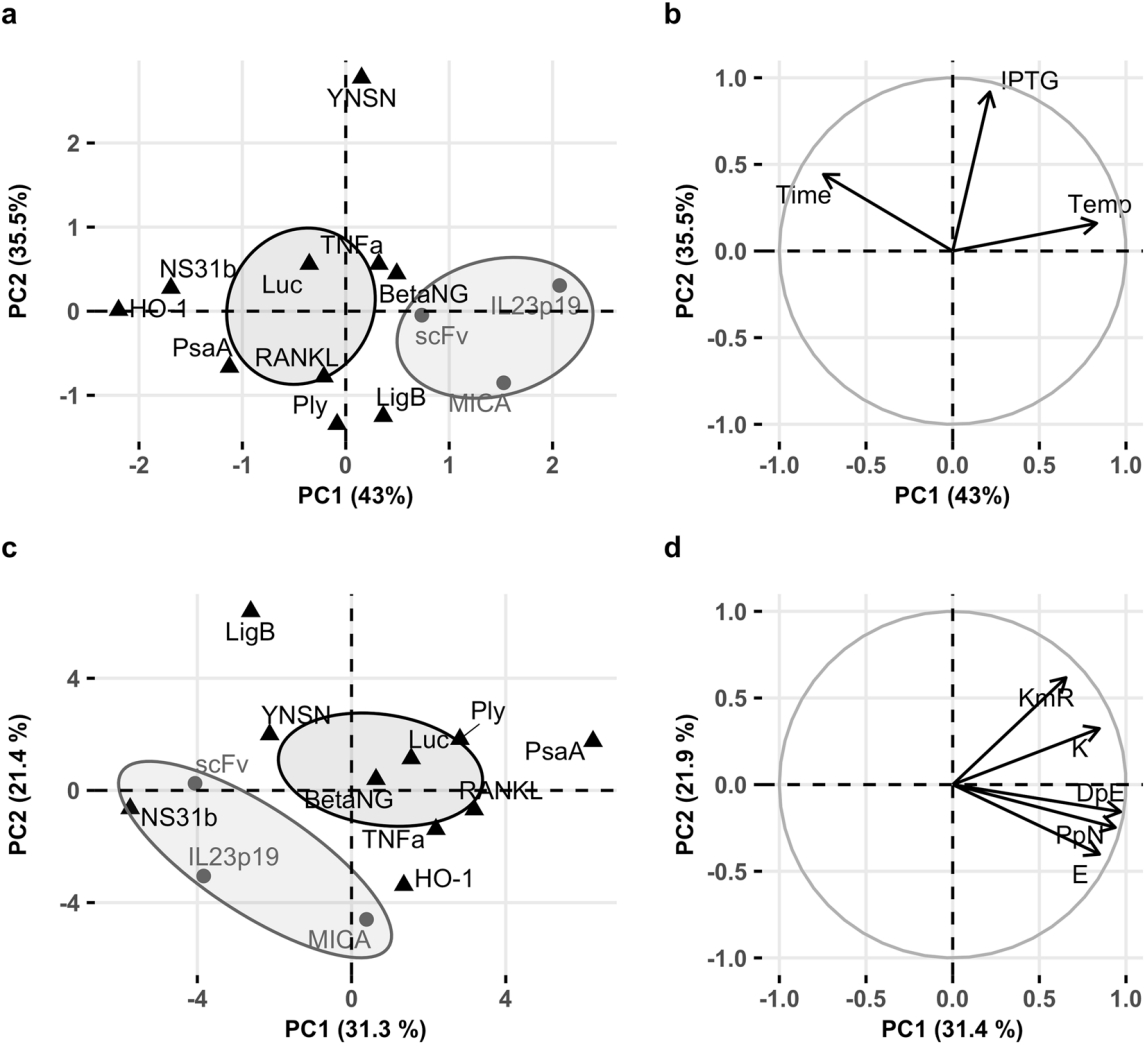
Principal component analysis (PCA) calculated from environmental (**a,b**) and sequence-derived features (**c,d**) for soluble and insoluble proteins. (**a**) PCA from environmental variables showing concentration ellipses for soluble and insoluble proteins at 95% confidence. (**b**) Correlation plot of variables used in (**a**). (**c**) PCA from sequence-derived features showing concentration ellipses for soluble and insoluble proteins at 95% confidence. (**d**) Correlation plot of variables used in (**c**). KmR: Lysine minus arginine ; DpE: Aspartic acid plus glutamic acid; PpN: Lysine plus arginine plus aspartic acid plus glutamic acid. Both analysis were performed on R using FactoMineR package.

Since different optimal expression conditions for each protein tested could be detected using our model, we decided to test whether there was any relationship between protein solubility and favored environmental factors by performing a principal component analysis (PCA). To this end, a literature search was carried out in order to select those publications in which the post-induction temperature, post-induction time and IPTG concentration were regarded as the optimal variables for bacterial culture, and whose numerical values for protein concentration were provided. We were able to select 10 reports (Table 3), which described different conditions for protein optimization. Interestingly, insoluble proteins from our work clustered together when plotting the first two principal components (78.5% of the total variance) (Fig. 3a). Furthermore, insoluble proteins were correlated with higher temperatures and lower expression times (Fig. 3b).

**Table 3:** Literature search of proteins optimized by a DoE approach.

Protein	Hosts tested	Expression vector	Tag	Design Type	Significance (p < 0.05)			Optimal value			Host	Type	Reference
					Temp	Time	IPTG	Temp	Time	IPTG			
NS3 1b L13K	BL21(DE3) BL21(DE3)pLys	pBEV11	His	Full Factorial and Box-Behnken	Yes	No	No	21	18	0.55	BL21(DE3)	Soluble	Swalley SE *et al*., 2006
PsaA	BL21(DE3) Star	pET28a	No Tag	CCD	Yes	Yes	No	25	16	0.1	BL21(DE3) Star	Soluble	Larentis AL *et al*., 2011
TNFa	BL21(DE3), BL21(DE3)pLys Rosetta	pGEX	GST	CCD	Yes	Yes	No	25	4	1	BL21(DE3)pLys	Soluble	Papaneophytou CP and Kontopidis GA, 2012
RANKL	BL21(DE3), BL21(DE3)pLys Rosetta	pGEX-6P-1	GST	CCD	Yes	Yes	Yes	25	6.5	0.3	BL21(DE3)pLys	Soluble	Papaneophytou CP *et al*., 2013
HO-1	BL21(DE3) Rosetta	pET28a	His	CCD	Yes	Yes	Yes	22	24	0.25	Rosetta	Soluble	Papaneophytou CP and Kontopidis GA, 2016
Ply	BL21(DE3) Star	pET28a	No Tag	Fractional Factorial	Yes	No	Yes	25	4	0.1	BL21(DE3) Star	Soluble	Marini G *et al*., 2014
LigB	BL21(DE3) Star	pAE	His	CCD	No	No	Yes	28	4	0.1	BL21(DE3) Star	Soluble	Larentis AL *et al*., 2014
Luciferase	BL21(DE3)	pET30a	No Tag	CCF	Yes	Yes	Yes	30	18	0.5	BL21(DE3)	Soluble	Islam RS *et al*., 2007
β-NG	BL21(DE3)	pET39b(+)	DsbA signal, His (Nterm and Cterm), S-Tag (Cterm)	CCD	Yes	Yes	Yes	25	2	1	BL21(DE3)	Soluble	Tilko P *et al*., 2017
non-specific nuclease	BL21, BL21 (DE3)pLysS StarTM (DE3)plysS	pET-24a and pET-24d	His	CCD	Yes	Yes	Yes	32	20.5	1.5	BL 21 StarTM (DE3)plysS	Soluble	Fang XJ *et al*., 2014

Besides environmental variables, we retrieved the protein sequence reported in the literature and constructed FASTA sequences. With this information, we derived 33 protein features normally used to predict protein solubility in *E. coli*, using a Biopython script^20,21^. We performed a second PCA with this information, observing again a separation of soluble and insoluble proteins into two different clusters (Fig. 3c). In this case, the protein NS31b, a protease domain from hepatitis C virus NS3 protein, was also clustered with insoluble proteins from our work^22^. In the PCA, components PC1 and PC2 explained 53,3% of the total variance, and the most important variables included amino acids content (K and E), and composite amino acids content K − R (KmR), D + E (DpE), K + R + D + E (PpN).

A correlation analysis between environmental variables and sequence-derived features revealed that there are a significant (p < 0.05), positive correlation between IPTG levels and cysteine, proline and the composite amino acid content K + R − D − E in the whole dataset (Fig. S2). No further significant correlations were found between these two types of variables.

### Optimization of refolding conditions

**Figure 4:**
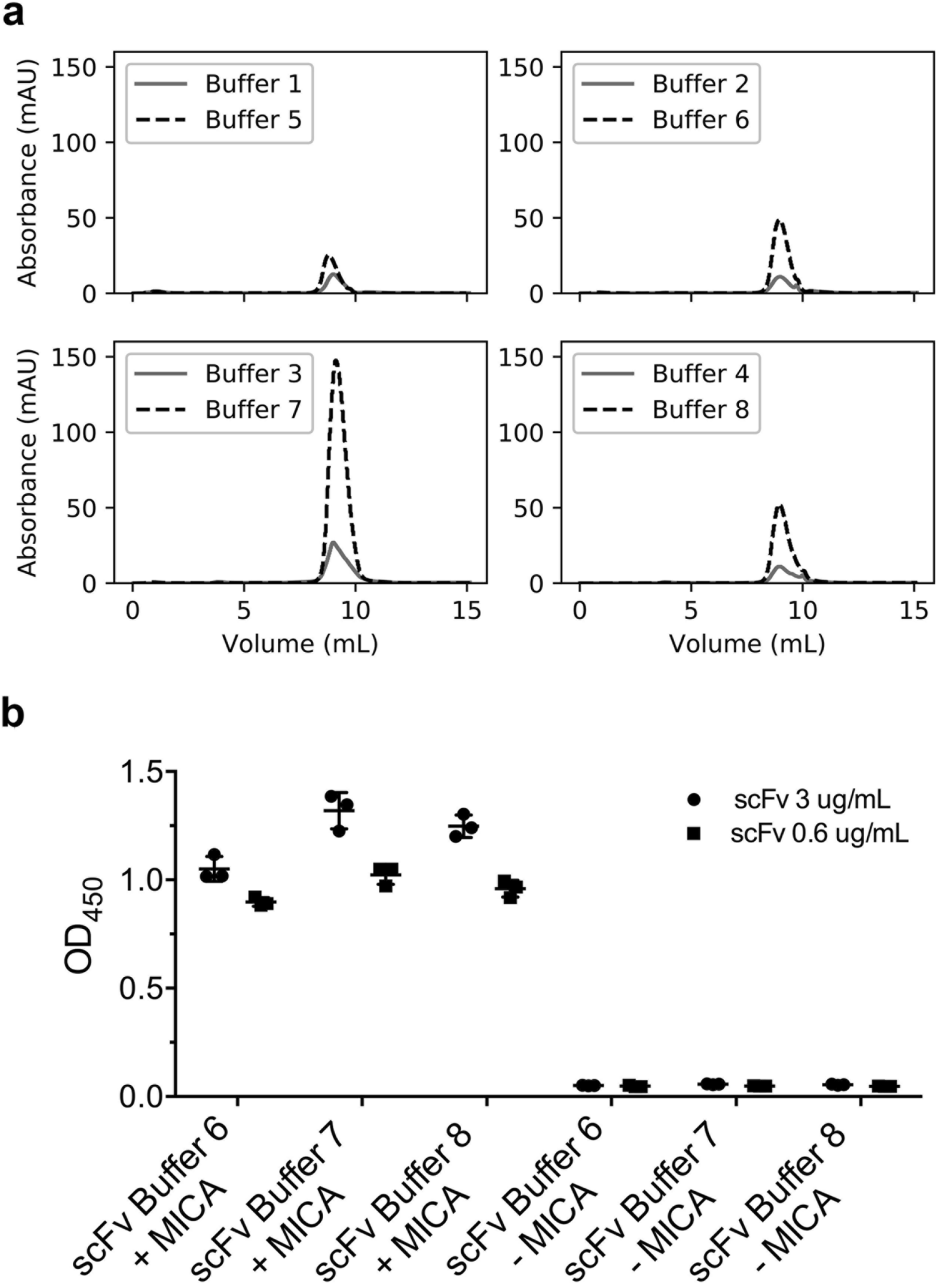
anti-MICA scFv renaturation experiments. (**a**) Ten microliters of purified anti-MICA scFv, in denaturing conditions, was fast-diluted in 200 µL of different refolding buffers. (**b**) The binding ability of purified and refolded anti-MICA scFv, in three different buffers, was analyzed by ELISA at two concentrations. Buffer 1: 50 mM Tris HCl, pH 7.4. Buffer 2: Buffer 1 + 500 mM NaCl. Buffer 3: Buffer 1 + 500 mM arginine. Buffer 4: Buffer 1 + 10% glycerol. Buffer 5: Buffer 1 + GSH/GSSG (10:1). Buffer 6: Buffer 2 + GSH/GSSG (10:1). Buffer 7: Buffer 3 + GSH/GSSG (10:1). Buffer 8: Buffer 4 + GSH/GSSG (10:1).

Although the DoE strategy adopted by us revealed the optimal conditions for protein expression, we still faced the challenge of a limited expression of anti-MICA scFv, which we attributed to the in-column refolding protocol (see materials and methods). Therefore, we decided to use a fast-dilution protocol, which provides the possibility to assess several buffers and additives. Refolding conditions were optimized using small-scale refolding assays in 96-well plate format. The chosen screening conditions were based on the literature and restricted to most probable positive conditions^23^. The success of refolding was tested by analyzing the soluble fraction of the protein by size-exclusion chromatography, as it retrieves information on the amount of protein and its aggregation state. As shown in Fig. 4a, the use of arginine and the redox pair GSH/GSSG (reduced gluthatione and oxidized gluthatione) resulted in the greatest increase in soluble protein concentration. Of note, all conditions with the redox pair showed a higher concentration of total protein, consistent with the presence of disulfide bonds in most scFvs^24^. Importantly, these conditions allowed us to obtain 1.1 mg/mL of anti-MICA scFv. The conditions were repeated with MICA, and the functional activity of the protein pairs was analyzed by ELISA. Our results showed that scFv, refolded in NaCl, arginine and glycerol (with redox pairs), was able to specifically detect MICA (Fig. 4b).

## Discussion

In this work, we employed a design of experimental methodology to optimize the production of recombinant anti-MICA scFv, MICA and IL-23p19 in *E. coli*. As expected, we found important yield differences at different environmental variables, with distinctive optimal conditions for each protein. In addition, for each protein studied, environmental variables had different effects on the generated models, reflecting some intrinsic protein properties that can affect protein biosynthesis and/or folding. Moreover, multivariate analysis showed that insoluble proteins were markedly different at the preferred environmental conditions and amino acid content from a set of soluble proteins described in the literature. Finally, we showed that a simple refolding experiment can be successfully applied to obtain the best conditions for full-scale protein refolding.

Obtaining high yields of soluble protein can be challenging, as the majority of proteins tend to aggregate as inclusion bodies^25^. This can delay a successful expression experiment, given the rather complex steps needed to obtain native protein in this scenario. However, inclusion bodies have several advantages, such as high levels of protein expression and purity. Optimizing the expression of recombinant proteins in this compartment, as we show in the present work, is relatively uncommon, although it can be a very promising strategy. The three-level Box-Behnken design is relatively easy to run with standard laboratory equipment. After a three-day experiment, including protein expression, inclusion bodies purification and solubilization, purification and refolding, a researcher can have complete working space and obtain important insights to develop a more robust and productive expression system. The yields obtained for each protein varied significantly, as expected; however, more importantly, they varied between each run. These differences should be interpreted as room for improvement, and to the fact that obtaining protein in inclusion bodies can be fine-tuned by different environmental variables.

Although there are other factors that can be optimized, including OD at induction, pre-induction time, the use of different plasmid constructions, the use of different hosts and codon optimization, we favored the speed of our analysis using factors widely reported in the literature^22,26–29^. Our main objective was to quickly find the most appropriate conditions for protein expression in order to perform further biological assays, and not to force the expression of the protein in the soluble compartment, which is not always successful and can significantly delay its production.

Multivariate analysis showed that soluble and insoluble proteins are distinct in their optimal environmental features and in their sequences. The expression of insoluble proteins correlated with higher temperatures and lower amount of time, probably reflecting the need to rapidly accumulate protein in inclusion bodies and avoid bacterial damage^29^. In the case of sequence-derived features of recombinant proteins, those that accounted for most variance were represented by the amino acid content (lysine, glutamic acid) and composites (Lysine minus arginine, DpE: Aspartic acid plus glutamic acid. PpN: Lyisine plus arginine plus aspartic acid plus glutamic acid). We expected more contributions from predicted features, such as length and absolute charge, which are regarded as the most informative characteristics for predicting protein solubility^21^. Interestingly, the protease NS3 appeared inside the concentration ellipse of insoluble proteins. Proteases tend to accumulate in inclusion bodies due to their toxicity to the host, which could explain this finding^30^. However, NS3 showed markedly different optimal environmental variables, suggesting that its optimal expression conditions cannot be predicted only from sequence features.

Our protocol for protein refolding was adapted from commercially available high-throughput methods. We favored a fast protocol, in which relevant conditions for protein refolding were assayed. In this respect, the most studied variables in the culture medium are NaCl concentration, since it can stabilize proteins by either hydration or exclusion of water^31^; the presence of arginine, which aids the refolding process, although its exact mechanism is still unclear^32^; and glycerol, which enhances hydrophobic interactions by ordering the solvent around the protein^32^. The use of the redox pair GSH/GSSG was considered as the scFv structures tend to have disulfide bonds^33^. We tested our protocol in scFv, since it is the most complex structure and showed the lowest expression yields between the three proteins studied. As expected, adding GSH/GSSG dramatically increased the yield of soluble protein, which was further enhanced by the addition of arginine. This experiment took only two days to complete, but offered invaluable information for further scaling the expression of scFv. More importantly, all conditions with additive/redox pair resulted in a similar ability to bind MICA, thus complementing our refolding chromatographic assay with a functional assay.

The use of statistical approaches for insoluble protein expression is a powerful strategy to improve the yield of recombinant protein. We successfully developed models to predict protein yields from inclusion bodies, which revealed that each protein has different requirements for the environmental variables tested. Moreover, the proteins tested in this work, which were expressed in inclusion bodies, showed a differed behavior in terms of sequence features and environmental variables for optimal expression, as compared with soluble proteins. Further protein processing, including refolding, was effectively applied to the most difficult-to-express protein in our set, using a simple approach with commonly used additives. The use of a redox pair is highlighted as a necessary strategy when disulfide bonds are suspected to be in the protein structure, as it dramatically improves the yield of soluble protein. Further work in insoluble proteins may unveil whether the behavior observed for this set of proteins is replicable, and potentially reveal optimal environmental factors for a given protein sequence, which will accelerate bioprocesses development and structural studies.

## Materials and Methods

### Design of experiments

A Box-Behnken design was generated in R^34^, using RcmdrPlugin.DoE package^35^. Three factors, which included temperature after induction, time after induction and IPTG concentration, with three levels each, were considered, accounting for 15 sets of experiments. This design was used to model the expression of anti-MICA scFv, MICA, and IL-23p19. Table 1 shows the coded and actual variables derived from the Box-Behnken design.

### Protein production

Proteins were expressed in BL21(DE3) bacteria as a HisTag fusion proteins. MICA was expressed as a truncated form using the extracellular domains α1 and α2^36^. Starting cultures were generated from pipette tip punctured glycerol stocks in 50 mL of 2xYT (BD Biosciences, USA) supplemented with corresponding antibiotics (ampicillin/kanamycin 50 µg/mL) (United States Biological). This bacterial culture was grown overnight at 37 °C under shaking (200 rpm). Next, 1 mL of starting culture was added to 200 mL of 2xYT/ampicillin/kanamycin and grown to mid-log phase (OD600 = 0.6). The bacterial suspension was then separated into 15 subcultures and protein expression was induced using IPTG. After this point, cultures were separated into their respective experimental runs. After protein expression, cell cultures were harvested by centrifugation, washed with ice-cold PBS, and resuspended in lysis buffer (25 mM Tris at pH 8.0, 100 mM NaCl, 5 mM imidazole, 1% Triton X-100, lysozyme plus protease inhibitors). This bacterial paste was stored at −80 °C until further processing. Bacterial lysis was carried out by sonication of thawed bacterial paste, on ice, with 8 cycles of 20 seconds and 40 resting seconds. Sonicated samples were centrifuged and the pellet (inclusion bodies) were harvested, washed once with washing buffer (50 mM Tris, 100 mM NaCl, 1% Triton X-100, 0.1% sodium deoxycholate (DOC), pH 8.0), and treated with denaturation buffer (50 mM Tris, 500 mM NaCl, 6 M guanidine hydrochloride, 5 mM imidazole, pH 8.0) overnight at 4 °C. In the case of IL-23p19, inclusion bodies were firstly incubated in washing buffer (50 mM Tris, 100 mM NaCl, 1% Triton X-100, pH 8.0) during 30 min at 37 °C and centrifuged. The resulting insoluble fraction was washed three times with the same buffer without Triton X-100 and treated with the anionic detergent N-Lauroylsarcosine (Thermo Fisher Scientific, USA) as denaturant agent (50 mM Tris, 500 mM NaCl, 1% N-Lauroylsarcosine, 5 mM imidazole, pH 8.0) for 1 h at 37 °C. Resuspended inclusion bodies were centrifuged; the supernatants were collected, filtrated through a 0.22 µm syringe filter unit (Advantec MFS, Japan), and loaded into a pre-equilibrated Ni-NTA matrix (Thermo Fisher Scientific, USA).

The purification process differed for each protein. For MICA, purification was carried out in denaturing conditions, washing with matrix denaturing washing buffer (50 mM Tris, 500 mM NaCl, 5 mM imidazole, 6 M guanidine hydrochloride, pH 8.0) and eluting with 6 column volumes of elution buffer (50 mM Tris, 500 mM NaCl, 300 mM imidazole, 6 M guanidine hydrochloride, pH 8.0). scFv was refolded in-column by serial exchange of matrix denaturing washing buffer to matrix washing buffer (50 mM Tris, 500 mM NaCl, 5 mM imidazole, pH 8.0). Refolded protein was eluted with 6 column volumes of elution buffer (50 mM Tris, 500 mM NaCl, 300 mM imidazole, pH 8.0). Finally, in the case of IL23-p19, purification was carried out in the presence of 0.2% N-Lauroylsarcosine. The concentration of detergent was brought to 0.2% by slow dilution of the sample and then applied to the matrix equilibrated in the same condition. Washing was performed with buffer containing 0.2% N-Lauroylsarcosine and 20 mM imidazole, followed by serial exchange of buffer until total dilution of N-Lauroylsarcosine. Elution was performed with 6 column volumes of elution buffer (50 mM Tris, 500 mM NaCl, 600 mM imidazole, pH 8.0).

Protein expression was assessed in bacterial cultures by SDS-PAGE. Each step of protein expression and purification was loaded into 10 % acrylamide gels. In the case of MICA and anti-MICA scFv, identity of the protein was confirmed by western blot, using a mouse anti-HisTag antibody, followed by anti-mouse IgG-HRP detection. Identity of IL23-p19 was confirmed by mass spectrometry using a MALDI-TOF/TOF 4800 Analyzer (Applied Biosystems, Framingham, USA).

### Protein renaturation

Denatured MICA fractions were pooled and refolded by rapid dilution in 100 mL of renaturing buffer (50 mM Tris, 500 mM NaCl, 3 mM GSH, 0.3 mM GSSG, pH 7.4) in order to achieve a protein concentration <10 µg/mL. Diluted protein was mixed overnight at room temperature. Next, the mix was filtrated through a 0.22 µm syringe filter unit and concentrated using 10 kDa Centricon centrifugal filters (Merck Milipore, Germany) to a final volume <1 mL. Renaturation was verified by size-exclusion chromatography in an Äkta Purifier FPLC with a Superdex 5/150 GL column (GE Healthcare, USA).

### Model generation

Data obtained from Box-Behnken design was fitted using rsm package^37^. The expression level of each protein was assumed to be influenced by the following independent variables: post-induction temperature, post-induction time and IPTG concentration. Model quality was assessed by r-squared values, lack-of-fit tests and normal Q-Q plots. Contour plots (conditions versus yield) were generated for each variable.

### Multivariate analysis

A literature search was carried out, in PubMed, in order to find optimized expression conditions for other proteins. The inclusion criteria were: (1) Protein was expressed in *E. coli*, (2) Protocols that were optimized through a DoE methodology including post-induction temperature, post-induction time and IPTG concentration, (3) Protein was purified and quantified and (4) Protein sequence was readily available in the work or through references. With this information, FASTA sequences were retrieved. Thirty-five features were predicted from protein sequences using a Biopython script^20^. Briefly, these features included amino acid content, 7 composites K − R, D − E, K + R, D + E, K + R − D − E, K + R + D + E, F + W + Y, length, pI, hydropathy, absolute charge at pH 7, fold propensity, disorder, sequence entropy, and β-strand propensity. All these features where selected from previous literature as potential indicators for protein solubility^21^. Data were stored as csv file and loaded on R^34^ for PCA analysis, using FactoMineR package^38^.

## Supplementary information

Supplementary Figures

## Data Availability

All of the data analysed during this study are included in this published article (and its Supplementary Information files).
