# Supplementary material for: Optimization of culture conditions for the expression of three different insoluble proteins in Escherichia coli
Source: Sci Rep. 2019 Nov 14;9:16850. doi: 10.1038/s41598-019-53200-7 (PMC6856375; doi:10.1038/s41598-019-53200-7)
Supplement: Supplementary file 1 — Supplementary Figures [file 41598_2019_53200_MOESM1_ESM.docx]

JOURNAL: Scientific Reports

TITLE: Optimization of culture conditions for the expression of three different insoluble proteins in Escherichia coli

Matías Gutiérrez-González^1,2^, Camila Farías^1^, Samantha Tello^1^, Diana Pérez-Etcheverry^3^ Alfonso Romero^1^, Roberto Zúñiga, Carolina H. Ribeiro^1^, Carmen Lorenzo-Ferreiro^3^, María Carmen Molina*^1^

^1^ Programa Disciplinario de Inmunología, Instituto de Ciencias Biomédicas. Facultad de Medicina, Universidad de Chile.

^2^ Programa de Doctorado en Farmacología, Facultad de Ciencias Químicas y Farmacéuticas, Universidad de Chile.

^3^ Área de Biotecnología, Instituto Polo Tecnológico de Pando. Facultad de Química, Universidad de la República Oriental del Uruguay.

*Corresponding author

E-mail: mcmolina@med.uchile.cl

Phone: +56 2 29786913





**Fig S1. Quantile-quantile plot of the three models generated**. **a**: MICA Q-Q plot. b: scFv anti-MICA Q-Q plot. **c**: IL23p19 Q-Q plot.


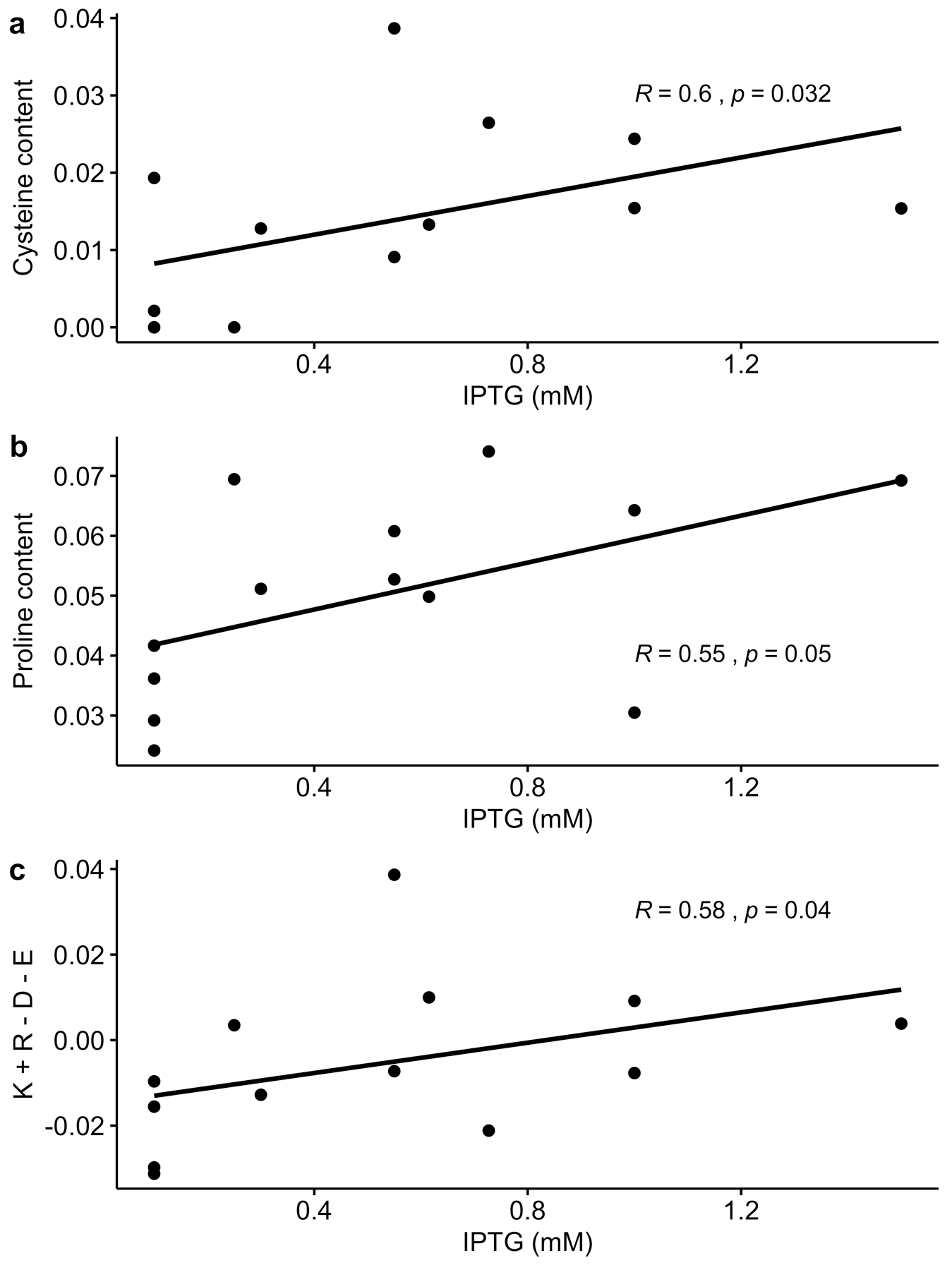


**Fig S2. Significant correlations between optimal IPTG concentration and sequence-derived features.** Significant (p<0.05) correlations between environmental and sequence-derived features are shwon in scatter plots. In the case of B, p = 0.049.

**Fig S3. Full-length gels and western blots from fig 1a and 1b.**

MICA protein expression is shown on the left. Anti-MICA scFv expression is shown on the right.
